# Observations on the Spread of Asiatic Cholera, and Its Communicable Nature

**Published:** 1849-09

**Authors:** John Evans

**Affiliations:** Prof. of Obstetrics and Diseases of Women and Children, in Rush Medical College


### ARTICLE X.

*Observations upon the Spread of Asiatic Cholera, and its Communicable Nature.*—By JOHN EVANS, M. D., Prof. of Obstetrics and Diseases of Women and Children, in Rush Medical College.

By the term *Communicable Disease*, I mean one that generates the poison by which it is propagated. If persons previously healthy, are taken with a specific disease when brought near those affected with it, or in contact with articles that have been exposed to them, and this shall appear generally to be the condition of its spread, it is reasonable to suppose it communicated to them. I prefer this term to contagion, because the literal meaning of the latter is too limited, implying transmission only by contact, while its general acceptation by the profession, implies certain conditions, such as a fixed or uniform time for its development–a febrile action in the disease--a particular mode of exposure to the affected individual–an exemption from second attacks—a certainty of infection from exposure—a uniform course for the disease to run, &c. The term infection is defined as synonymous with contagion, and by Dunglison its meaning is restricted to cases of actual contact.

Another reason why I prefer the term Communicable, is, that the idea of whole cities and neighborhoods being contaminated by the morbific agent, may be thus introduced, which would be difficult by the use of the terms above referred to.

If the minds of my readers can be divested of the necessity of all the conditions above referred to; and be directed to the simple fact of communication from those affected with the disease to others, without any prescribed rules for that communication, (which should be the first step in the investigaton) we shall be able to furnish some evidence that is worthy of consideration.

As to the horror excited in the minds of some, on account of the moral and social influences of the doctrine of communication "the hurried and barbarous interment of the dead, scarce waiting till the breath had left the body—the worse than brutal desertion and neglect of friends and relations—all growing out of a belief in the contagious nature of the disease," I have but a word to say and a question to ask. Let us know the truth and risk the consequences. Whether is it more humane to lead our friends and relations into an exposure of their lives, by which thousands upon thousands shall be sacrificed to their temerity, if the disease is communicable; or to allow them to exercise their fears while there is doubt as to the contagion?

It is remarkable, that in the observations made upon cholera by the profession, in different parts of the country, we so often find hypotheses laid down to begin with, and facts hunted up to sustain them. Thus the Miasmatic, Animalcular, Cryptogamous, Atmospheric, Telluric, Electric, Ozonic, Carbonic, and other theories are advanced, and in the minds of the authors, doubtless proved conclusively by the *facts* thus adduced. And all this is done in an age when Medical Philosophers boast of reasoning by the inductive system---" in a century that flatters itself that it is the Nineteenth."

That it is extremely difficult for the mind to remain without forming conclusions, during the observation of the phenomena of disease, until the subject is thorougly investigated, is evident from the chaos of exploded theories that have always obstructed the path of the medical enquirer. But the conclusions to which I am forced, by this history, were made in spite of a prepossession against the doctrine of contagion, formed by receving opinions of authors, aside from a careful examination of the mode of its progress and invasion of different countries and places.

The observations of physicians in India where the disease is indigenous, should not longer be the authoritative history of its character, as it appears among us on this side of the globe.

It is because the opinions of Aunesly, Johnson, Searl, Jamison, and others, who have observed it in India, have been looked to, instead of a careful annotation of its nature and mode of spreading among us, that we find so many of our authors treating as absurd, the doctrine of its communicable nature.

I shall confine my remarks principally to the disease as it occurred in this country, and especially in the Mississippi Valley, and in Chicago and its vicinity, during its recent invasion.

But first, let us glance at the mode of its introduction into this country in 1832. About the first of June, of that year, the cholera made its appearance for the first time on our Continent.* The Carricks, a vessel direct from Ireland, where the disease was prevailing when she left, arrived at Gross Island, the quarantine station for Quebec having lost 29 persons of cholera on her passage. On the 8th, a case occurred at the quarantine; on the 9th, several cases occurred at Quebec wharf, most of which were from on board the steam-boat Voyageur, which had taken passengers from on board of emigrant vessels below, and started for Montreal, but on account of bad weather, landed about 200 in Quebec. The first cases

*Dr. Morrin, Health Officer at Quebec.

in the city were nearly all of this company. In that part of Quebec where they were landed, the disease first broke out and committed its greatest ravages.

The Voyageur proceeded to Montreal. One passenger died on board of Cholera, on the passege. The first two cases in Montreal were from this boat. Dr. Morrin says, after stating these and other facts, "All things considered, I am satisfied the cholera is contagious."

The disease certainly spread about as rapidly as the travel on the Lakes along the Canada shores of Lakes Ontario and Erie; quarantine regulations at the ports on the other coast having turned the travel principally through Canada.

After this it soon made its appearance on steamboats on Lake Champlain. In about the usual length of time of travel for emigrants by land, many of whom were on the route, from here to New York, the disease broke out in the latter city. Thence it travelled along the Hudson, breaking out first at Albany, then at the larger towns on the western thoroughfare; and soon it became prevalent, first in the large cities, as a general rule, and afterwards spread to the villages and into the surrounding country, few places escaping.

But my purpose is to speak more particularly of its recent invasion of the country, as the facts are more accessible, and the evidence of its communication more pointed.

For a clear and minute history of its progress from its ancient focus around Jesore on the delta of the Ganges in different directions through Asia, during this its second spread from that hot-bed of pestilence over the civilized world, we would refer to an article by Prof. Dickson, in the New York Medical Journal for January last, derived principally from Dr. Verollot, Physician to the French Embassy at Constantinople. This history shows its transportation, with scarcely a variation, along the thoroughfares of travel, which vary in direction to almost all the points of compass.

The following extract from the London Medical Gazette, shows the result of the observations of its Editor on the progress of the disease in Europe:

"It is worthy of remark, that in 1830–'2, as in 1847–'8, the cholera has manifested itself chiefly in the great lines of intercourse along frequented roads, and the banks of navigable rivers, attacking chiefly towns and cities where the population was most dense, producing the largest amount of mortality in its first onset, then slowly diminishing in severity, and finally disappearing to reappear in a neighboring locality. According to Dr. Lasegue, the greatest rapidity with which the cholera has spreah over any locality has not exceeded a rate of from 250 to 300 miles a month. This comparatively slow progress, together with its advance in the face of prevailing winds, is very unlike the usual mode of diffusion of a purely epidemic disease."

On the 2d of December last, the ship New York from Havre, France, with a large number of passengers, (21 cabin and 331 steerage,) principally French and German emigrants from various parts of Germany,* and from Paris and Havre, arrived at the quarantine ground on Staten Island, 8 miles from the city of New York. Nine days previously the cholera broke out among the steerage passengers, and several died before the arrival, when eight or ten cases were taken from the vessel to the quarantine store houses. With some irregularity, the disease prevailed on Staten Island, and particularly in the Marine Hospital, until January 4th, when it entirely disappeared. The mortality in proportion to the population, at this place, was great. One person from the quarantine was taken with cholera in the city, but was sent back and died.--- Another case took it and died in the same house where he was taken.

*A writer in the New York Courier and Enquirer, says many of these emigrants were from Hamburg and other parts of Germany where the disease was prevailing when they left, and "one or more of these poor people brought with them the bedding or clothes in which one of their friends had died of cholera." The weather had been mild until reaching the raw atmosphere of the Grand Banks, where the disease broke out, "when open comes the big chest, and out comes the infected clothing."

No other cases occurred for 9 days, when a German, who had had no traceable communication with the infected places, took it and died. This was all of the disease that occurred in the city, and it disappeared entirely from the quarantine establishment and hospital.

We observe in the report of Dr. Whiting, Health Officer, that those exposed to the persons affected, seemed particularly liable to take it.

Although this sudden arrest of the disease within such a short distance of New York, without its spreading into the city, was in the winter, it should not be contended that this fact, aside from the quarantine, was sufficient to account for the circumstance, or else why did it prevail to so great an extent at Staten Island in the month of December. The season of the year may have rendered it more amenable to this kind of control, for its whole history shows that it spreads less rapidly in cold, than in warm weather.

The history of its spread up the Mississippi Valley affords a better opportunity of investigating its communicable nature, than has been before presented; since it had but one point of departure, N. Orleans; a healthy country over which to spread, and this at a season when the travel from N. Orleans on the river was great, and the conveyance rapid and direct for thousands of miles; while through the country generally it was but little and difficult, on account of the bad roads of winter. Whereas, the former invasion of the country was made when there was much travel in all directions, and several of the great marts, as Quebec, Montreal, New York, &c., were early affected and of course the channels of its communication soon intersected each other, so as to become confused and lost.

At New Orleans, on the 11th of December last, 9 days after the appearance of cholera at Staten Island, the ship Swanton arrived from Havre with two cases on board. At the time of the departure of both this vessel, and the New York from Havre, there was no cholera at that point. But it is worthy of remark, that passengers on both these vessels were from Germany, where the disease was raging, and that the disease made its appearance in parts of France as early as October last, though it had not reached Havre or Paris when these vessels sailed. Seventeen persons died on the Swanton during her passage, the disease having broke out when she was about two weeks at sea. At N. Orleans no quarantine regulations prevented the Swanton from landing at once in port, and the cases on board were taken to the great Charity Hospital, where, up to January, as many as fifty cases occurred among the nurses, and persons admitted for other complaints.* Several cases appeared soon after among the shipping at the landing, but were only regarded as aggravated cholera morbus. From this time the disease spread rapidly. The deaths for the week ending on the 15th, 4 days after the arrival of the Swanton, before which no case had been reported, were 17. During the next 5 days there were 87 deaths, after which the disease produced a fearful mortality, assuming the form of a general epidemic; the number of deaths up to the 4th of January inclusive, as reported, being 1115. From this time the disease rapidly disappeared, so that on the 6th of February the city was said to be entirely free from it; but it reappeared soon after, and produced a much greater mortality.

From a very early day after the disease spread so rapidly among the shipping in the port of New Orleans, where an immense fleet of steam-boats are continually arriving and departing, it began to prevail on board of these vessels while lying there and on their upward passage on the Missisippi and its tributaries.

It extended rapidly up the river, but it is remarkable, that along its course, the points having the most communication with the steam-boats, were the first to be infected, and in

*Dr. Fenner in N. Orleans Med. Jour.

no instance were any of these points affected until steam-boats from places below, where the disease was prevailing, had arrived.*

The disease extended up Red River from point to point in the same manner as up other tributaries of the Mississippi.

At Memphis, Tenn., a case occurred on the 22nd of Dec., in a boy who had been employed in huckstering fruit, &c., about town and the wharf, where he was in the habit of selling to the steam-boat passengers. Previous to this, Dr. Shanks says* there had not been any striking tendency to diarrhœa, more than usual in such damp rainy weather, although the boy had had it for several days previously. Steam-boats from this time were continually passing up the river, that reported cases and deaths on board, but for 4 days no other case occurred, when two cases at a considerable distance from each other, and from the steam-boat landing, occurred on flat-boats. The disease now rapidly spread, the whole line of flat-boats being about two miles in length, the population on which was near one thousand, but Dr. Shanks thinks, under circumstances that preclude the idea of its spreading from one to another directly, yet he believes it communicable through the air. These numerous cases occurred before the disease extended to other parts of the city, the boats being separated from the city by a wide batture.

Dr. Buchanan, of Nashville, Tenn., says,† that on the 20th of January last, 20 days after the first boat from New Orleans with cholera on board arrived, and after several other boats had been there, upon which cases of cholera occurred, one case of the disease appeared in a house almost surrounded by water, in the lower part of the city. It was a man of bad habits who had exposed himself to the weather, and in the water for several days. The next case was in the same family.

*Shanks on Cholera in American Jour. for July 1849.

†West. Lancet for Feb.

After the boats from New Orleans, on which there were cholera patients, commenced arriving at Louisville, we hear of the occurrence of the disease there, but have not been able to obtain the date of its first appearance. Various other points on the Ohio River, soon after reported cases.

Cincinnati, it will be borne in mind, is the point where a large portion of the passengers are discharged from steam-boats that ascend the Ohio River from New Orleans, and we would therefore expect that the disease would, if communicated, be found here at an early day after it broke out in New Orleans. Accordingly we find on the 27th of December, Prof. Harrison giving a clinical lecture over two cases at the hospital, which came from steam-boats that had brought them up the river.* Whether these were cholera or not, it was soon prevalent in Cincinnati. This was 12 days after it was prevalent among the shipping, and on board of steam-boats departing from New Orleans.†

Here the disease was slight, presenting only the form of diarrhœa, excepting occasional cases, until about the first of May, when it assumed the more aggravated form, from which time, until in July, it prevailed, and in June, with great mortality. The deaths up to Aug. 10th being 4,114.

About the 12th of May cases of the disease began to be reported along the Little Miami and Mad River Rail Road, upon which the travel generally goes from Cincinnati to New York.

The disease soon made its appearance at different points along this great thoroughfare of travel. On June the 7th, five cases had occurred at Buffalo, three of which were attacked on board of vessels, and were from Chicago and Cincinnati, where the disease was prevailing when they left.‡ In the mean-time it was carried up the Ohio River and broke out at various

* Western Lancet.

†The time occupied in the trip ascending the river from New Orleans to Cincinnati, is from 6 to 8 days.

‡Buffalo Med. Jour. for June.

points, thus travelling the other great thoroughfare to the Eastern Cities.

Boats arrived at St. Louis with cholera on board on the 27th of December. On the 28th the Amaranth, with 30 cases on board landed there. On the first of January it had become epidemic in rather a mild form. The following from the St. Louis Medical Journal for March, gives the general facts of its introduction into that city:

"Since the 1st of Jan. up to the present time, 67 deaths have been reported as occurring from cholera. Of these, at least one half were persons who contracted the disease in New Orleans, or on their way from that city to this, and who were landed here in an almost dying condition. The remaining cases have been of local origin. During the same period, 47 cases have been admitted into the St. Louis Hospital—of which, 20 have died, and 27 recovered. Of the 20 fatal cases, a large majority were admitted either from boats or obscure and filthy parts of the city, in a state of complete collapse, from which it was impossible to arouse them."

The disease almost subsided, only an occasional case occurring, so that in February the city was thought to be clear of it. In the early part of April, (26 cases on the 10th,) the disease increased; the warm weather seemed to aggravate the cases and favor the development of the specific poison. In addition to which, the increased influx of the disease from below added greatly to its spread.* It increased until in June the mortality was as great as has been the fate of any American city; the deaths having reached the number of 180 in one day, in a population, at that time, about 40,000. Seventeen practitioners of medicine fell victims to the disease. Just before the disease subsided, a rigid quarantine stopped the emigrants from landing in the city.

*To give some idea of its great prevalence on boats ascending the river from New Orleans, where it had brok out with greater violence than in the winter, we give the following items from the Cario Delta of March 29th: Steam-boats arrived on 22d March—the Bride reported 7 deaths on board; the Uncle Sam 3 cases. On 23d the Belle Key 8 cases; the Gen. Washington several cases and 1 death; the Seraph 8 cases and 2 deaths; the Gladiator 8 cases and 3 deaths. On the 24th, the Tennessee 3 cases; the Yorktown 10 deaths.

By an intelligent gentleman, a citizen of St. Louis, who recently passed through this place from there, I learned that the greatest mortality was in the highest and most pleasantly situated part of the city.

From St. Louis it extended up the Missouri River, and broke out among the emigrants to California at their rendezvous at St. Joseph; great numbers of whom had passed through and started from St. Louis during the months of March and April.

The Sacramento arrived at St. Joseph with one case of cholera on board on the 21st of April, and on the 12th of May it was reported that boats continued to arrive with cholera on board. One vessel, the Mary, had buried 36 passengers on her trip up from St Louis, and the disease was said to be prevailing to an alarming extent among the emigrants.

The disease also spread up the Mississippi river. Five cases were reported to have occurred at Quincy, Ill., on the Upper Mississippi, on the 20th of March.

At Galena, about the middle of April, the first cases occurred in a family that came up the Mississippi on a steamboat, four of whom died.

On the 18th of March the Illinois River opened, and the steam-boat trade commenced. About the 1st of April, two cases of cholera were reported at Peoria, said to have come up the River. There had been none of it previous to this time, but soon after it appeared at other points on the river.

On the 20th of April the Illinois and Michigan Canal was opened, so that boats could pass through, and the line of communication was complete from St. Louis through it and the Illinois River to Chicago.

On the 29th of April the canal boat John Drew, arrived here with a number of emigrant passengers on board, who were direct from New Orleans, by way of St. Louis, several of whom were sick, but whether of cholera or not, I cannot ascertain. They immediately left the boat. The captain of said boat, Mr. J. Pendleton, was taken sick on the same day. He was seen by Drs. Myers, Stewart, and others. The disease soon run into the collapse stage of cholera, and he died on the night of the 30th.

This is the first case of which I can get an authentic account. Others were soon reported in different parts of the city. Its greatest ravages for a considerable time were near the river, but as canal boats were almost hourly arriving, and no pains were taken to observe them, the manner of its spread cannot be traced. It is possible that from this time a stream of "*cholera atmosphere*," which soon prevaded different parts of the city, continued to flow in by way of the canal. Emigrants too were arriving by the Eastern route, from the 20th of April, when the lake navigation opened, many of whom were from various parts of Europe, where the cholera was prevalent, and from the quarantine at Staten Island, where it again prevailed from and after the first week in April.* The health of the city up to this time had been as good as usual at that season of the year.

That part of the city in which there was the greatest mortality, and in which scarcely an individual escaped an attack, either in the form of a diarrhœa, or the more aggravated disease, is a neighborhood of three squares, in the North Division, situated on the highest ground in the vicinity of Chicago. The soil is very sandy and dry. These blocks are thinly built up, and are nearly surrounded by open, vacant ground. The inhabitants are mostly of the better class of Norwegians, in moderate circumstances, who live as comfortably as the average of Americans. The three blocks numbered 332 inhabitants. Of these, 44 died of choler

The disease had prevailed in other parts of the city for two months before this neighborhood was affected—only one case having occurred in one of the inhabitants, a Swede, who was taken sick in a distant part of the city on the 5th of June, and

*New York Jour. Med. for July.

speedily recovered. From this time, no more of the disease occurred until the 7th of July. A day or two previous to this time, thirteen emigrants from Sweden, direct by way of New York and Buffalo came into a house occupied by Samuel Arns, and a family of 8 persons. One of these emigrants took sick of cholera on the night of the 7th of July, and died, under homœpathic treatment!! the next day. These Swedes had been unpacking their chests of clothing the day before the man above referred to took sick. After his death, the rest of the emigrants were turned out of the house, and most of them left the neighborhood.

The day after the death of the Swede, there were four of the members of Arns' family taken down. Of these, Mr. Arns' mother-in-law and his child, died, but the others recovered.

Andrew H. Nelson, who lived in another part of the same house with three other members of this family, all of whom had been attending upon, and rubbing the Swede, were taken down on the day he died, and survived but a few hours.

Notwithstanding the fearful mortality in this house, up to this time the health of the neighborhood had been good, with the above exceptions. On the night of the 14th of July, a young woman was taken, one block South of Arns' house, on the same street, and died on the 15th. No intercourse with the sick could be traced, except that she had been passing Arns' house on the side walk. Swend Olson, who lived in the next house to Arns', had a slight diarrhœa for a day or two—attended upon the young woman above referred to, until she died, was taken with the disease immediately, and died the next morning, July 16th, at 2 o'clock.

Of the family in which Olson died, Mrs. Gunwald, Mrs. Olson, and a young woman died also—one on the 23d, and one on the 24th of July, and the other on the 5th of August.

Mr. Gunwalson, who was the captain of a vessel in the harbor, sailed at once for Michigan City with his wife for fear of the disease. They however returned in health on the 25th of

July, and found the disease still in their house. Mrs. Gunwalson was taken the next day after their return, but recovered. The Captain was at home when Mrs. Olson died in his house, on the 5th, and leaving his wife sick of cholera, sailed again for Michigan City on the 7th, where, on the 9th, he was taken with it, and on the 10th died.

From Andrew Nelson, Janitor of Rush Med. College., I obtain the following—

On the 31st of July, Knuit Hanson, nursed Nels. Nelson until he died, which was about noon. At 2 o'clock on the same day he took a diarrhœa and went to Andrew Nelson's: on the 3d of August he died. Nelson's family were in tolerable health up to this time. On the 4th one of his children was taken with the disease. Two days afterward another child and a sister were taken. On the same evening another sister was taken with diarrhœa, but was relieved by medicine taken at the onset of the attack. On the night of the 9th Mrs. Nelson, who had been washing up the cloths of Hanson that died the day before, and nursing her children, was taken violently and died the next evening. On the morning of the 10th Mr. Nelson's brother was taken and died before night. Two members of the family, only, escaped an attack.

Many incidents of a similar character occurred in the neighborhood, but we forbear to relate more of them lest they become tedious.

A circumstance worthy of remark, is, that when a patient was bad, a large number of the neighbors were sure to crowd into the house, as few of them had any fears of the communicable nature of the disease.

Mr. N. H. Ellickson, resident in the neighborhood, has taken the census of it, and made out the following table:

A large part of those who are not marked as exposed in the table, had been either living with, or in constant intercourse with those who were attending upon, or visiting cases of cholera. There were also several taken who went into houses to live after the cholera had been there and subsided. In such cases, it is possible that the fomites remaining had not affected those in the house, because at the time not susceptible, but others coming in contact with it were poisoned. In the same manner, those living in the house, becoming susceptible, might be taken. Such phenomena frequently occur in the course of infectious diseases.

*Table showing the number of those exposed---The time between exposure and attack, and the duration of the disease in those who died in three blocks in Chicago:*

| Names of those who had the Cholera. | When taken. | When died. | Had been directly exposed. | Time from exposure to attack. |
|---|---|---|---|---|
| Emigrant from Sweden, | July 7th | July 8th, | unknown. | |
| Ann Christine, | " 8 | " 27 | 1 | 1 day |
| Mrs. Arns, | " 8 | " recovered. | 1 | 1 " |
| Arns' Child, | " 8 | " — | 1 | 1 " |
| A. H. Nelson, | " 8 | recovered. | 1 | 1 " |
| Miss Belsey, | " 14 | " 15 | unknown. | |
| Saml. Linbla, | " 15 | " 16 | 1 | 7 days |
| Chas. Linbla, | " | recovered. | — | |
| Swiend Olson, | " 16 | " 16 | 1 | 6 hours |
| Johanna Norboe, | | " 19 | unknown. | |
| Sever Lewison, | " 16 | " 20 | 1 | 36 hours |
| Lewis Knuitson, | " 19 | " 19 | unknown. | |
| Mrs. Hymen, | " 20 | " 20 | 1 | 1 day |
| Hymen's Child, | " 20 | " 20 | 1 | 1 day |
| Mrs. Mayors, | " 19 | " 20 | unknown. | |
| Mayors' Child, | " 20 | " 20 | 1 | 1 day |
| Mrs. Tiffny, | " 21 | " 23 | 1 | 3 days |
| 3 English persons, | | " 20 | 3 | uncertain |
| Mrs. Olson. | " 21 | " 22 | 1 | 7 days |
| " Rohlgel, | " 21 | " 22 | 1 | 1 " |
| Ann Gunwaldson, | " 21 | " 23 | 1 | 36 hours |
| Mrs. Peterson, | " 22 | " 23 | 1 | 7 days |
| Ann Swenong, | " 23 | " 24 | 1 | 8 " |
| Mrs. Abraham, | " 24 | " 25 | 1 | 1 " |
| N. Nelson, | " 26 | " 27 | unknown | |
| T. Elliason, | | recovered. | " | |
| Sever Olson, | " 26 | " 27 | 1 | 23 hours |

| Names of those who had the Cholera. | When taken. | When died. | Had been directly exposed. | Time from exposure to attack. |
|---|---|---|---|---|
| Sever Amonson, | July 18 | recovered | 1 | 12 hours |
| Mrs. Amonson, | " 19 | " | 1 | 1 day |
| J. A. Balkin, | " 30 | July 31 | 1 | 1 day |
| C· Knuitson, | " 31 | " 31 | 1 | 2 days |
| S. Cederbery, | " 30 | Aug. 6 | 1 | uncertain |
| Ann Breynelson, | " 31 | | 1 | 1 day |
| Knuit Hanson, | " 30 | " 3 | 1 | 12 hours |
| Mrs. Balkin, | " 31 | recovered. | 1 | 8 hours |
| Ole Oddson, | " 25 | Aug. 1 | 1 | uncertain |
| Saml. Cederbery, | Aug. 1 | " 7 | 1 | 1 day |
| Mrs. Ellickson, | " 1 | " 5 | 1 | 1 day |
| R. Johnson, | " 2 | " 4 | 1 | 1½ days |
| Mrs. Olson, | " 3 | " 5 | 1 | uncertain |
| Mrs. Liberg, | " 6 | " 7 | Norwegian emigrant. | |
| " " Child, | " 6 | " 7 | " | " |
| T. Michaelson, | " 6 | recovered. | 1 | 1 " |
| N. Christopherson, | | " | 1 | 2 days |
| Mrs. Malin, | " 7 | " 8 | 1 | 12 hours |
| — McKeef, | | " 10 | | |
| Mrs. Nelson, | " 9 | " 10 | 1 | 1 day |
| Peter Nelson, | " 9 | " 10 | 1 | 1 " |
| Gnuder Johnson, | " 10 | recovered. | 1 | 1 " |
| L. Nelson, child, | " 10 | " 10 | 1 | 1 " |
| Dinah Abraham, | " 11 | recovered. | 1 | uncertain |
| Mrs. Michaelson, | " 11 | " | 1 | 5 " |
| Ragneld Nelson, | " | " | | |
| S. Michaelson, | " 12 | " 12 | 1 | 1 day |
| A. Knuitson, | Aug. 13 | Aug. 14 | 1 | 1 " |
| Mrs. Knuitson, | " 13 | recovered. | 1 | 1 " |
| K. Anderson, | " 14 | " | 1 | 1 " |
| K. Lawson's child, | " 18 | " 18 | 1 | uncertain |

### SUMMARY:

| | | |
|---|---|---|
| Whole number of cases observed, - - - - - - - | | 60 |
| Deaths, - - | 44 | |
| Recoveries, - - | 16 | 60 |
| Directly exposed to the disease, - - | 50 | |
| Exposure not traceable, - - - - - | 10 | 60 |
| Of those exposed there elapsed before the attack, over 24 hours in - - - - - | 11 cases. | |
| Twenty-four hours in - - - - - | 25 " | |
| Less than 24 hours in - - - - - | 6 " | |
| Time uncertain, - - - - - - - | 8 " | |
| Not known to be directly exposed, - - | 10 " | 60 |
| Of those who died, there were sick one day or less, - - - - - - - - - | 28 | |
| Over one day, - - - - - - - | 16 | 44 |

In this table all cases are omitted unless of decided character, and only those are put down as exposed who had been visiting or nursing the sick of cholera; but most, if not all of those not marked exposed, had been with persons direct from the cholera chamber, or in houses where cholera patients had recently been sick. The emigrants came by way of New York and Buffalo, a few days before taken, where the disease was prevailing when they passed. It shows the length of time between exposure and attack to be generally about one day, which strictly corresponds with the history which follows.—Also, that the course of the disease generally runs about the same length of time, when it proves fatal.

Many incidents that occurred in other parts of the city strongly point to the communicable nature of the disease, a few of which are given. The following is from Dr. J. E. McGirr, of this city:

Mr. Blakie, an Irishman, who had been attending on some friends who had the cholera, was taken with the disease on the 25th of June, Mrs. Phalen, who lived in the next house to Blakie's, and her son, were taken and died on the next day. While going to the burying of her mother and brother, Miss Phalen, who had nursed them during their sickness was attacked with cholera on the 27th, and unable to go home, was taken to the house of Drs. P. and J. E. McGirr, where she died on the 29th. Her brother, who had also been with his mother and brother when sick, while attending upon her, was taken on the morning of the 29th at 7 o'clock, and died the same evening. At 12 o'clock the night after Miss Phalen died, in the same house, Dr. McGirr's little girl, who had been in the room with Miss Phalen while sick, was taken with the disease. Mrs. McGirr, after nursing the little girl all night, was taken on the morning of the 30th, and in the evening of the same day the doctor himself was attacked. The last three recovered. Three members of the family escaped.

The following history of the manner of its communication from one to another through an extensive family of connexions, several of whom lived in remote parts of the city from each other, was also furnished me by Dr. J. E. McGirr, who attended upon them all:

Richard Welch, who lived in the north part of the city, died of cholera on the 20th of June. Mrs. Neagle attended upon him while sick. On the 6th of July one of her children died with the disease, and on the 8th another. On the 11th, Mrs. J. Welch, who had been nursing Neagle's children, died.—Her nephew, Mr. Maloney, who was with her and had been to see Neagle's children while sick, was taken on the 11th, but recovered. His mother, in the same house, took the disease on the 12th, and died on the 15th of July.

Mr. Dunnovan had been nursing Mrs. Welch, who died on the 11th. Her child took the disease on the 13th, but recovered.

Mrs. Dunn, sister of Mrs. Welch, attended upon her while sick, but lived in the South part of the city. Her child took the disease on the 15th, four days aftei, and died the next day. Another child took it on the 16th, and died the same day in the evening. Mrs. Dunn took the disease the next morning and died the same day. The same day, Mr. Dunn, her husband, was taken, but recovered. Mrs. Hannebury, sister to Mrs. Dunn attended her while sick on the 17th, and had a child taken on the 18th, but it recovered.

Dr. Brainard furnishes me the following incidents:

An Irishman and wife, who had recently emigrated to this place and were living on Michigan Avenue, died of the disease. The woman was taken while washing the clothes they brought with them, on the 3d of August, and died the next day. The man was taken on the 4th, and died on the 7th. On the 5th, Rev. Mr. Patterson visited the man several times during the day, returning to his family. On the next day his child was taken with cholera, but recovered. These Irish people being poor and friendless, Mrs. —, kindly attended upon them both, and superintended the funeral arrangements of the man on the 7th, passing frequently to and from her own house. On the morning of the 8th her child was taken with the disease and died the same day.

These instances of its transmission through whole family circles and neighborhoods, in direct succession, with but an occasional variation, might be extended to any desirable number, were it not that they become tedious by extensive multiplication, and we let the above suffice.

But the introduction of the disease into the country around Chicago furnishes the best evidence of its communication that has yet been afforded.

The City is situated so that on the East lies the broad watery expanse of Lake Michigan. On the West is a wet prairie almost uninhabited for ten miles; and on the North and South the sandy lake shore is so closely bordered by the extension of this wet prairie that the country is very thinly settled. An occasional tavern for the accommodation of travellers, constituting the principle habitations.

Thus, it will be seen, that the city is almost isolated on all sides.

When the cholera became prevalent, the intercourse with the country was almost suspended. Those who live here, however, will bear me testimony that the winds blow freely and frequntly in all possible directions, so that there could scarcely be said to be any obstructions to the free and rapid passage of "*cholera atmosphere.*"

A circumstance at my office enables me to say positively that during the months of May and June, the winds were from the East to the West a considerable part of the time. Of this a stove pipe, a little distance from my East window, frequently and faithfully reminded me.

By reference to a table in a subsequent part of this article it will be observed that at the time the cholera began to prevail extensively in the country west of Chicago, about the 1st of Aug., the winds were never from the east.

Under these circumstances, if the disease was atmospheric in its origin, whether the poison be animalcular, cyptogamous, carbonic, or malarial, we should expect the rich and densely populated country beyond the surrounding prairie, to be speedily brought under the baneful influence of this worse than Simoom after the disease broke out in Chicago. But the history of its spread here, as elsewhere, shows it to correspond with the amount and rapidity of human intercourse.

Instead of its accustomed 300 miles per month, we find it prevaling here for two months before any number of cases occurred in the county, and it was not until the disease had been here three months that the county became generally affected. By physicians from the country who visited the city during this remarkable exemption from cholera; I was informed that the health was unusually good there, for the season of the year. But the manner of its introduction into the different neighborhoods of the surrounding conntry, forms the strongest link, in the history of the spread of the disease, for the support of the doctrine of communication.

James Kerr, resident a few miles distant, and well acquainted with the persons referred to, and Dr. Maxwell, of this city, have furnished me with the following account of the occurrence of the disease at Summit 12 miles South-west of Chicago on the Ills. and Mich. Canal.

This neighborhood had been quite healthy, and no case of the cholera had occurred among the inhabitants up to this time.

On Sunday, the 17th of June, a man came from Chicago to remain for a few days, on business, at Mr. Heacocks, was taken with the cholera and died the next day.

Mr. Heacock was taken with the disease and died on the 23d. A son on the 24th; Mrs. Heacock, another son, and an Irish girl, who had gone from Chicago to nurse them, died on the 25th. Another son was also taken very bad at this time but recovered. A daughter who was taken and brought to the city, recovered.

Mr. Webster, who assisted in nursing and burying the last of the above who died, lived at Esq. Brown's, in the neighborhood, was taken on Tuesday the 26th and died in a few hours.

On the next day Esq. Brown was taken and died on the 28th. On the evening of the same day, Mrs. Brown who came to Chicago, was taken as she entered the city and died early the next morning. Mr. Guthrie, her father, and Mr. McGlashen, her brother-in-law, while taking the corpse to Summit for burial, in a close carriage, were both taken while crossing the prairie. Mr. Guthrie survived a few days and died, Mr. McGlashen recovered.

Thos. Ferrity was at Mr. Heacocks, while the disease was producing its awful ravages in the family, nursing the sick, he went home at night unwell and told the family, with whom he lived, to leave the house for he was going to have the cholera. The family obeyed his warning, and the next day he was found in the house dead and alone. The family that fled escaped the disease.

Mr. Bankson, who lived near Ferrity's, and about a mile from Heacocks, had also been there while they were sick, took the disease, and died the same day. Mrs. Bankson was attacked about the same time. Mr. Grant, another neighbor, was at Heacocks rendering assistance to the sick, but escaped. His son, who attended the funeral of some of the family, however, took the disease soon after and died. These were all the cases that proved fatal, and the neighborhood became quite healthy by the 2d week in July.

Mr. Kerr observed that but two or three of those who rendered the kind offices of assistance to the sick during this terrible scourge escaped.

Dr. Knapp, who visited them professionally and remained with them sometime, had a severe attack immediately after.

The neighborhood ten miles west of this city at Doty's tavern, at the terminus of the plank road, on the great western thoroughfare from the city, has thus far escaped; but one case having been brought there fiom town, which was not admitted, but sent back to a house in the suburbs, where he died.

The following history of the appearance of the disease in the country near Aurora, on Fox River, 40 miles west of Chicago, was communicated to me by Mr. Favour, apothecary and student of medicine of that place:

The first case that occurred was about five miles west of Aurora, in the person of a pedlar who was direct from Chicago. He stopped at Mr. Sanford's, a farm-house on the road, on Wednesday the 27th of June, and died of cholera on the following Sunday. On Monday Mr. Sanford was taken with the disease, but recovered. Several members of his family were soon after taken with diarrhœa, but all recovered speedily, and no other case occurred in the neighborhood or in the surrounding country, until the 22d of July, when Mr. Van Fleet, a farmer living three miles north of Aurora, returned from Chicago, where he had been to market, and was taken with the disease the same night. He died the next day. On the 24th, the day after he died, Mrs. Van Fleet, who attended upon him, was taken and died in 18 hours after the attack. On the 27th, a brother of Mr. Van Fleet, who lived within a short distance of his house, was taken. This was all of the disease that had occurred in this neighborhood, which was otherwise healthy up to that time.

About five miles distant from Mr. Van Fleet's, and eight miles from Aurora, a Mr. Leech was taken with cholera on his return home from Chicago, and died on the 24th of July. His son was subsequently taken. These were the first cases in that neighborhood.

The following account of the appearance of the disease in two neighborhoods, is furnished by Dr. D. W. Boyd, of Brush Hill, who attended upon the cases referred to. The Buck

Horn tavern is 15 miles north-west of Chicago on the Milwaukee road, and is kept by the doctor's brother.

On the 3d of July, Mr. Biddle, who lived two miles beyond, was taken with cholera on his return from Chicago, and could get no farther than Mr. Boyd's tavern. After nursing him 2 days, Mr. Boyd was taken with the disease on the 6th.

Soon after five others, being all of his family, were taken with diarrhœa. All these recovered, being early put under the influence of calomel, camphor, and opium. The neighborhood, so far as the Dr. was able to learn, and his brother's family in particular, had been healthy up to this time.

At Flagg Creek, twenty miles a little south of west from Chicago, the disease made its first appearance on the 22d of July, under the following circumstances:

Mrs. Martin, a citizen of Lockport, 35 miles south-west of Chicago, on the canal, came to the city on the 18th of July, and remained three days. She was taken with a diarrhœa on the 19th, and on the 22d went to Mr. Helmick's at Flagg Creek settlement. At 10 o'clock that night she was taken with rice-water discharges and died at 10 o'clock the next morning. On the 27th, four days after this case died, J. T. Webb, a sailor, came from Chicago to Mr. Glaton's who lives, about 80 rods from Helmick's. He had a diarrhœa when he left Chicago, where he saw a patient sick with cholera. He was taken bad on the 27th, and died on the 28th of July. Mr. Helmick, who assisted in nursing Webb, was taken with diarrhœa on the day he died, and on the 29th had a severe attack of cholera, but recovered.

Mr. Craigmile, who also assisted in nursing Webb, was taken with a diarrhœa on the day he died, and on the 30th it was a well developed cholera, but he recovered.

On the 31st a Norwegian, who lived at Galton's and assisted in nursing and burying Webb, was taken with cholera and also recovered. He had been to see Craigmile, the case above referred to, the day before.

On the 3d of August, a German, who lived at Craigmile's above referred to, was taken with cholera, but also recovered.

In reference to this history, Dr. Boyd remarks: "Previous to the above cases the neighborhood had been very healthy.—Diarrhœa now became general in the families of Helmick, Craigmile, Glaton, and others adjacent, excepting two families who lived on the Carrington farm, about three-fourths of a mile distant from Helmicks, the members of which kept away from the cholera patients. Diarrhœa continued to 17th of August and subsided. It was easily managed by the use of calomel, camphor and opium."

The Dr. relates the case of a Mr. Bush, living at the house of Mr. Farwood, who had not been exposed (?) that occurred on the 26th of August, a day or two before he wrote, which he considers a victim of fear. (If fear produces cholera, ought we not to meet with cases continually?) But, says the Doctor, the man had been in a rage because a girl came to live in the family, from a house where two men had died of cholera.

The circumstances of its appearance at Naperville, 30 miles west of Chicago, were furnished me by my colleague, Prof. Brainard, who, a few days ago, returned from a visit to that place.

Some weeks before any case of cholera occurred there, tests for ozone became quite dark. About the 22d of July, an Irishman, by the name of O'Brien, returned from Chicago, where he had been visiting a relative who died of cholera.—Soon after his arrival at home he took the disease and died. In about three days after, a German girl, who had been working at his house, took the disease and died on the 26th. Dr. Sowers attended upon the girl, and took the disease and died the next day. Dr. Hough, attended upon Dr. Sowers—took the disease and died two days afterward, about the 20th of July. Miss Rouse, who lived at Dr. Sowers, remained at his house about 10 days for fear of carrying the disease with her, packed up her clothes, went home to her fathers, three miles in the country, and soon took it. About the 17th of Aug. her father was taken with the cholera, and died on the 20th. Mrs. Rouse, his daughter-in-law, who had been with him while sick, took it and died on the 23d. Her husband took the disease and died the same day. A son-in-law, who lived in Naperville, was there attending upon the sick above referred to, took the disease on the 37th, and died in a few hours.

A man who had lately lost his wife and child by cholera, came to Naperville, and died of the disease at the Pre-emption House on the 24th. Mr. Snyder, who roomed with him, took it died the next day. Mr. Mackintosh, who boarded at the same house, took it and died on the 26th. Up to and during this time, the country around it, and the village containing about 600 inhabitants, enjoyed good health, with these exceptions.

Dr. Kimberly, of this City, furnishes me with the following account of the disease as it occurred in the neighborhood of Cazanovia, 15 miles west by north-west of Chicago; where nine persons died in rapid succession; the neighborhood having been healthy up to the time mentioned.

About the 20th of July, Mr Barnum came to Chicago to attend the funeral of his brother, who died of cholera. He returned home in the evening after attending the funeral, was taken with the disease the same night, and died in a few hours.—Three of his neighbors, who visited him while sick, were taken immediately afterwards, and died in a short time after their attack.

In the Dutch Settlement, near Mr. Barnum's, the disease was introduced by a German, who was taken the night after going from Chicago, a short time after the fatality above referred to, and died in about 12 hours. Four others, who visited him while sick, were taken soon after, and all died within four days. These were all the fatal cases that occurred, and the disease soon entirely disappeared.

Dr. J. W. Freer, of Wheeling 20 miles north-west of Chicago, writes as follows:—

"Below you will find a statistical account of the cases of cholera, that you desired me to report; I have given you the exact facts as they occurred in regular order. But there was several other cases of mere symptoms that seemed to arise from the same sources, which I thought not worth while to report. For instance, I myself had the symtoms premoniory to cholera, and several other young men who attended upon the sick.

"Now does it not seem that the young German was the starting point from which the disease radiated; certainly his exposures were sufficient, although his case was not one of pure cholera. Mr. Bruce was the only person who had been exposed in Chicago, but you will observe that he was the third in order of attack, providing the two first were in part cholera.

"Aug. 17th. German, name not known—had diarrhœa a week previous to the attack; pulse full, rather hard; headache; skin warm, but moist; griping tenesmus,—discharges resembled washing of meat; cramping in the legs; vomiting was excessive, attended with intense thirst.

"Had been to Ohio in the height of the cholera season. Habits very irregular, having lived here and there, as it happened. Had been to Mr. Culvers 2 or 3 days. Discharged Aug. 20th convalescent.

"Aug. 20th. Child of Mr. Culvers, age 3 years---symptoms similar to the case above; considered both cases as dysentery and cholera mixed. The child did not cramp as much as the German, but the vomiting was more profuse. Cholera symptoms ceased soon, but the dysentery continued 10 or 12 days.

"Had been away from home for several days, at a relations. Returned 2 days previous to attack, had eaten green trash at will. Had had slight diarrhœa for several weeks. Discharged Aug. 30th convalescent.

"Aug 20th. Mr. Bruce---taken in the evening with diarrhœa; color light towards morning; vomited, and purged rice-water discharges; cramped severely in every limb and abdomen. Saw him at 7 o'clock A. M. in that state; skin as if par-boiled; tongue cold, and nearly pulseless at the wrist; urgent symptoms ceased in about two hours.

"Had been at Chicago 3 times during the cholera season; The last time about a fortnight before the attack. Had been boarding and working at Mr. Culvers several days previously ate his last meat there—a man of steady habits, had eaten scarcely any vegetables. Discharged Aug. 24th.

"Aug. 21st. Mr. Culver—had been subject to diarrhœa for two or three days previous to the attack, which was about two o'clock A. M. Commenced with the characteristic discharges of cholera, with violent cramping. Found him in that state at 7 o'clock in the fore noon. Remedies seemed to have no effect upon him.

"Had not been in Chicago during the cholera season. Family had lived almost exclusively upon vegetable diet. Had had a villainous stew of pot pie 2 or 3 days before, from which he and the family dated their sickness. Died at 11 o'clock A. M., same day.

Aug. 22d. Mrs. Culver, mother of Mr. Culver—diarrhœa, slight vomiting; yielded to treatment without the acute stages of the disease coming on.

Had been at home continually during the epidemic. Discharged Aug. 24th.

Aug. 24th. Mrs. Culver, wife of Mr. C.—Cholera symptoms the same as the old lady.

Same habits of living, had not been away. Discharged Aug. 27th.

Aug. 25th. Jas. Alexander—Cholera symptoms; yielded to treatment at once.

Aug. 24th. Mr. Buskirk—symptoms nearly the same---some slight cramping; recovered in two days.

The following extract of a letter from my colleague, Prof. McLean, dated August 22d, gives an account of the disease as it appeared in Jackson, Mich., the place of his residence, a town of 3,000 inhabitants, situated on the Michigan Central Rail Road:

"Thus, we passed along with but few diseases, and less than the usual number of deaths, until the latter part of July, at which time we had a number of deaths in quick succession; all by cholera, and under the following circumstances:

"On Tuesday, the 24th of July, Mr. and Mrs. Cooper, two very aged persons, arrived here from the State of N. York. On their way they remained a number of days in the city of Detroit, and in that part of it where the cholera was prevailing at the time. On Thursday the 26th, two days after their arrival there, the old gentleman, after having had the premonitory symptoms a short time, was violently attacked with the disease and died on the following day, and the body was not interred until the next, nearly 24 hours from the time of death. During this period, the family, consisting of 8 persons, remained and slept in the house, which had some four or five different apartments. Sunday morning, July 29th, (the day after the body was interred) the old lady was taken down with the cholera; and also, about the same time, a son, and then one after another of the family in quick succession, and in less than 48 hours they were all dead, excepting one small girl, who, on Sunday morning, was sent away to one of the neighbors. While the others were sick and dying, another of the family, a young lad about twelve years of age, was also sent away from home; but when in the street, was taken with the disease, and carried to a neighboring house, where he died within the day. The woman of this house became much alarmed, and left, but the man remained at home. On the following day he undertook to clean and white-wash the room, and slept in it, alone, over night. The next day, about 10 o'clock A. M., he was taken very suddenly with cholera. This case yielded to treatment.

"During the fore part of the same week, a son of Mr. Bennett, who lived on the opposite side of the street from that house where the mortality was so fearfully great, was attacked with the disease, and died on Wednesday. This young man had not been with the other cholera cases, although he lived close by, but his father had been with them much of the time, in consequence of which the young man was very much alarmed.

"The number and suddeness of these deaths created quite an excitement in town, and but very few visited the sick, and many left the place. None of the attendants were attacked with the disease, unless it be that the diarrhœa, which some of them had soon after, might have terminated in it, had it not been checked.

"Mr. Cooper's family did not reside in an unhealthy part of the town—they lived in a neat frame house—were in comfortable circumstances, and cleanly in their habits; and the same may be said in regard to Mr. Bennett's family.

"It is now quite healthy, and in fact has been so all Summer. We have not had a death by cholera since that of Mr. Bennett. All the deaths were confined to those two families, nine in all, eight out of one, and one out of the other. The weather so far, this month, has been cool and pleasant; and ever since the occurrence of those cases, the town has been remarkably healthy.

"I have frequently experimented for the purpose of ascertaining the condition of the atmos phere in regard to Ozone, but at no time discovered any marked indications of its presence."

I have been at great pains to collect the foregoing history, and in all my enquiries have not met with a single instance in which the di ease made its appearance in a neighborhood, except there had been communication with persons or places affected with it.

I have been particular throughout to give names and dates that the correctness of my statements may at any time be investigated—a precaution deemed necessary in all such investigations, but more especially so in this case, where under the most favorable circumstances for observation such a mass of testimony is presented.

And this is the history of the spread of cholera as bearing upon the doctrine of its communication. First, it originates in India. From thence it travels over Asia and Europe, following the lines of human intercourse. It twice pursues the same general course, and twice appears upon the American coast, and in 1848 at two points nearly simultaneously. It stops its ravages under quarantine regulations at the one, and without them extends rapidly at the other. In all these instances of its appearance in the country, it breaks out immediately after the arrival of ships from Europe with cholera on board. It extends from New Orleans up the vast extent of the Mississippi and its tributaries, never appearing at a place until a vessel arrives from the seat of its prevalence with the disease on board.

It confines itself for months strictly to the banks of these streams, in its first appearance through the vast region of country that they meander. It spreads to the interior from these river towns with a rapidity proportioned to the intercourse with the places where it first makes its appearance. It prevails in Chicago for months before it extends to the country around it, where there is an interruption in the intercourse with the country and a city isolated from the settlements around it. It spreads to the surrounding country only as persons have intercourse with the city during its prevalence. It extends through different neighborhoods in the country only as far as the communication with the sick is kept up, and rapidly subsides in all small communities. As if on account of its rapid communication those susceptible to its influence at the time, are speedily taken, and for want of others to take it, it entirely disappears. It commits its greatest ravages in particular neighborhoods, and in Chicago, where the intercourse among the inhabitants is most intimate and frequent.

In sixty persons affected in one neighborhood direct communication can be clearly traced in fifty, and indirect in nearly, if not quite, all of the remaining ten.

In most cities its greatest prevalence is transferred from one neighborhood to another. In a large number of instances the disease can be traced as communicated from one to another through whole family circles. And during its subsidance the fact has been remarked that the cases that lingered in our midst in this place, generally occurred in houses where the disease had been before, and often affected those engaged in washing up the clothing and cleaning the sick chamber, as if they might have come in contact with the fomites of communication.

Notwithstanding neighborhoods of limited extent seem to be brought under the influence of the poison, do not these facts show conclusively that the disease is communicated? That it is self propagating? That in its action on the system it developes the morbific agent by which it is extended to others? Certainly no other reasonable explanation can be given of its course and progress.

The principle objection to the doctrine of contagion in cholera, is found in the exemption of persons exposed from attacks of the disease; a circumstance which I believe is entirely overated, as most persons in cities where the disease is prevalent, feel more or less of the symptoms of the disease, generally a sense of contortion in the abdomen, with or without diarrhœa. At least this has generally been the report given, and especially of those much among cholera patients. In such cases the powers of nature or early medication as is generally resorted to by nurses and physicians, seems adequate to resist and throw off the poison. We know that the contagion of all diseases, meets with resistance from the idiocyncracy of numerous individuals. Now if one or two persons in a community, by this cause, may be exempt from the contagion of one disease, may not half, or two thirds of them be in like manner fortified against the poison of another?

When five out out of six persons exposed to the coatagion of syphalis, scabies, or gonorrhœa, do not take the disease, (and this proportion is by no means uncommon) does any one deny that the sixth contracted it in that way? Again, would not the exemption weigh equally against a cause in the atmosphere? Yes! more forcibly, for in such a cause the whole city or community must be immersed, and consequently exposed.

And it is said that it often appears where no communication can be traced. How many of the assertions to this effect are gratuitous, I cannot say, but I do know that in numerous instances where they were made in this community, a little investigation proved the contrary to be the fact. And it is very consistent that it should be so, for it is easy to assert that no communication was known, and it requires much time and patient investigation to ascertain the facts of each case.—But the difficulty of tracing the communication, would, if admitted as valid argument, prove the non-contagious nature of nearly all diseases. Prof. Dickson* says he attended in the winter of 1847–8, forty patients attacked with small-pox, "not one of whom had ever seen a case, or consciously visited an infected neighborhood."

Aside from its being communicated by inoculation, and the fact that fewer people are naturally fortified against its contagion, there would be more difficulty in proving variola contagious than cholera.

I am satisfied that the communication in cholera can be more certainly traced than in rubeola or even variola, because a less

*Amer. Jour. Med, Sci. for July, 1849.

time elapses between exposure and attack. This rapidity of the action of the poison on the other hand makes it by far more difficult to follow the disease in its diffusion, for it will seem to appear simultaneously in distant parts of a city at a very early day after its introduction. The rapidity of the disease too, may account for the greater prevalence at the outset and gradual subsidence, as those acutely susceptible are speedily affected, while in the decline only those take it who subsequently become proper subjects.

And again it is asked, if cholera be communicable, why do not the physicians take it? In reply to this, I may refer to the fearful mortality iu our ranks by the pestilence, which has laid low some of our brightest ornaments; and to the fact that almost all of us have suffered from the early stage of the disease. I have no doubt that there are more cases of cholera among physicians in proportion to their numbers, than any other class of persons, but as they are constantly on the alert for the first symptoms of the disease, and understand the dread penalty of neglecting treatment, they generally do not allow it fully to develop itself. Notwithstanding this, more than half of my professional acquaintances in this city have suffered quite severe attacks of the disease during its recent prevalence.

Cholera is said not to be contagious because persons are liable to second attacks, and the disease can be interrupted in its course. Now are not the diseases referred to, (syphilis, scabies, and gonorrhœa) of undoubted cotagious nature, and liable to the same objections? Most certainly. And if they were not, have the objections truly any force? No! It is because we continually try to study one disease by looking at another, that we so often run astray. One specific disease can furnish us no data from which to judge of the nature of another. It is because cholera is a specific disease, and differs from any other, that it needs to be studied by actual observation, not by comparisons; and because all diseases are modified by climate, the habits of the people, avocations, and regimen, they require to be studied in the varying conditions and circumstances under which they occur in different countries and regions of country.

But it is urged that quarantine regulations, and sanitory cordons, are not competent to arrest the disease. This does not seem to be true in all instances, for Pittsburgh in 1832 and 1849, New York in 1848, and other places of smaller importance, would seem to indicate that at least some good has been effected by them. But admitting it to be generally true that they are inefficient, it would require that these regulations be conducted upon the system of complete non-intercourse, which I believe has, as yet, never been tried, before their failure could constitute a reasonable argument against the doctrine of communication.

But let us glance at some of the various theories that have been advanced in reference to the cause of cholera, and see how far each is entitled to consideration, and which will best explain the mode of propagation of the disease.

*Atmospheric Theory.*—It has been the fashion in all ages of the profession, where the mysterious nature of a disease was such as to leave the observer in ignorance of its cause, rather than acknowledge the humiliating fact, to forthwith attribute it to some changed or unhealthy condition of the air. Particularly in reference to all epidemics, is the atmosphere made the scape-goat to bear off the sins of our ignorance, and most admirably does it subserve the wanted purpose, for who can prove that it is not contaminated—that there is not floating in it the fatal miasm? Who can prove a negative, at least until some evidence is given to combat? Certainly no one; and in this consists the strength of the doctrine of the atmospheric origin of cholera. Has any one detected a change in the character or composition of the air, to which might be attributed the baneful influence? No! Has any one offered any other evidence of the doctrine, than that people take the cholera while breathing? If so, I have not seen or heard it; and the same argument might equally well find the poison in in the influence of the moon or the stars.

To explain the spread of cholera by the atmospheric theory, requires us to *suppose* in the first place, that the air is contaminated. Then that this contamination is self-propagative by fermentation, vegetation, spawning, or otherwise, or else the poison would soon become so diluted as to run out. Then that this poisoned air travels regardless of the course of the winds.—That small bodies of it may go to great distances in very narrow channels.—That a streak of it crosses the ocean three thousand miles from Europe to America, and strikes at a single point, and invariably that point a sea-port town.—Then that it will tarry a month at the quarantine within eight miles of New York city, without going farther, and depart for parts unknown.—Then that it will travel the whole length of the navigable waters of the Mississppi and its tributaries without varying to the right or to the left, and that it will hover over ships, steam-boats and caravans as they journey on their way. And finally, that like contagion, it follows individuals in their journeyings to the interior, and where they stop it for a time, hovers immediately around them.

After having made all these suppositions to found its existence upon, would it not be folly to attempt by the same, or any other kind of reasoning, to show this miasm to be either animalcular, cryptogamous, or gaseous?

*Telluric Emanations.*---In reference to the telluric origin of the disease, I have only to say that it is a strange freak of Mother Earth that sets her to giving out her poisonous emanations in such long and narrow streaks as mark the course of the cholera up the Mississippi and its tributaries.

*The Electrical Theory.*---Electricity has become a formidable rival to malaria, in reputation for the production of disease.

But as evidence in reference to its effects are more tangible, and its existence cannot be doubted, the probabilities are strong that it will not long be able to sustain the unequal contest.---But it is in reference to elecrrical disturbances causing the cholera, that we propose to speak.

Audrand communicated to the Academy of Sciences, of Paris, on the 10th of June last, that he had proved that the cholera depended upon the absence of the proper amount of electricity, as his machine refused to give its sparks in clear weather during the prevalence of cholera in Paris, but that the machine, upon the decline of cholera, again threw out brilliant sparks, "it might almost be said, with delight, as if aware of the good tidings it was bringing." This is the strongest evidence, of the only positive kind, that has been afforded to this theroy. From this single result of testing, he concludes as follows: "The question now appears to me entirely solvod." In Russia, and other places, similar experiments have resulted in the same way. Dr. Bell,* of London, finds in the electrical nature and condition of the food used by cholera patients just before attacked---the subsidence of cholera after storms, and the working of the electrical machine, ample maierials for the explanation of the production of cholera. Now ought not every one to know that the electrical machine is very irregular in its action, even when there has been no cholera for years? And storms by no means uniformly have the effect of lessening the disease. And do not our omniverous people eat food of all possible electrical qualities every year? Certainly, this kind of reasoning, or rather jumping at conclusions, is a fair specimen of the *post hoc, ergo propter hoc*. But it is said that the cramps and nervous twitchings may result from the action of electricity. Most certainly they may, but in a far greater measure from doses of strychnine.

The reasoning in favor of the electrical theory of cholera may be very learned, and indeed is, to a great extent,

*Newspapers.

excessively so, but the evidence is too small in its favor, and too great against it, to entitle it to very serious consideration. However, as it has attracted so much attention from the savans of the East, we cannot pass from the subject without giving it further attention.

Is not the electrical communication between all parts of the world almost instantaneous? Then why have for a month at Staten Island, the fatal absence of the fluid, while it is all right in the city of New York? And why have the disease occupy one year in extending from St. Petersburgh to Chicago? Might not the cholera be produced at any time, if absence of electricity caused it, by insulation and exhaustion? But in all the experiments with the machine since its invention, we hear of no case having been produced, even of cholarine.---We find the telegraph wires faithful in announcing from its very seat the intelligence of its greatest prevalence. Nor have we been able to learn that they do not work as well during the prevalence of cholera as formerly.

As bearing upon this point, we introduce the following table, by which it will be perceived that, as has been said by Prof. Olmstead, the electricity during cholera is subject to the same influences as at other times---moisture interfering with, and dryness favoring the evolution of the fluid. It will be observed that about the time of the greatest mortality in Chicago, the machine gave its longest sparks; longer, the gentleman* said who worked it, than he had seen produced by it at any former time.

The table, which was made up from the record kept at the Chemical Laboratory of Rush Medical College,† under the direction of Prof. Blaney, and the reports of the City Board of Health, is interesting, as showing the correspondence between the conditions of Electricity, Ozone, the Winds and

*Ferris Deming, Assistant in the Laboratory.

†Porf. Blaney informs me that he has instituted a series of experiments and observations, of which this table will be a part, for ascertaining the relation between tests for ozone and disturbances in the electrical conditions of the atmosphere, &c.

Weather, and the prevalence of the cholera. As ample thermometrical statistics are on record from other places that bear upon the point, they are omitted.

It may be well to observe that during the period included, the temperature has been generally moderate, and the weather unusually fine and uniform for this place. However, at the time of the great increase in the mortality, about the 20th of July, the weather for a few days only, was excessively warm and sultry:

*Table showing the length of the Electrical Spark on the Machine —The Test of Ozone---The course of the Winds---Character of the Weather, and the Bill of Mortality from Cholera each day for thirty-five days during its greatest prevalence in Chicago.*

| *Date.* | *Length of Electrical spark.* | *Indication of Ozone, S. side of building.* | *N. side of building.* | *Winds.* | *Weather.* | *Mortality.* |
|---|---|---|---|---|---|---|
| July 25, 7 A. M. | none | | | | | |
| " M. | none | light | light | light | damp and | |
| " 7 P. M. | none | | | northerly | rainy | 11 |
| July 26, 7 A. M. | none | | | northerly | rainy | |
| " M. | $\frac{1}{8}$ inch | medium | medium | | clear | |
| " 7 P. M. | $\frac{1}{4}$ inch | | | | clear | 18 |
| July 27, 7 A. M. | $\frac{3}{8}$ inch | | | | | |
| " M. | 2 inch | medium | dark | northerly | clear | 20 |
| July 28, 7 A. M. | 2 inch | | | | | |
| " M. | 1 inch | dark | dark | southerly | clear | |
| " 7 P. M. | 1 inch | | | | | 14 |
| July 29, 7 A. M. | none | | | | cloudy and | |
| " M. | none | very dark | very dark | | rainy | |
| " 7 P. M. | none | | | | thunder | 18 |
| July 30, 7 A. M. | 1 inch | | | | | |
| " M. | $2\frac{1}{2}$ inch | medium | medium | westerly | clear and | |
| " 7 P. M. | $2\frac{1}{2}$ inch | | | | dry | 14 |
| July 31, 7 A. M. | $2\frac{1}{4}$ inch | | | | clear | |
| " M. | 3 inch | medium | medium | southerly | dry | |
| " 7 P. M. | 2 inch | | | | | 25 |
| Aug. 1, 7 A. M. | $1\frac{1}{4}$ inch | | | | | |
| " M. | 3 inch | medium | medium | southerly | clear and | |
| " 7 P. M. | 3 inch | | | strong | dry | 21 |
| Aug. 2, 7 A. M. | $2\frac{1}{2}$ inch | | | | | |
| " M. | 2 inch | medium | medium | southerly | clear and | |
| " 7 P. M. | 1 inch | | | | dry | 15 |
| Aug. 3, 7 A: M. | $\frac{1}{2}$ inch | | | | dry | |
| " M. | none | medium | medium | southerly | rain, thunder | |
| " 7 P. M. | $\frac{1}{8}$ inch | | | | clear | 9 |
| Aug. 4, 7 A. M. | $\frac{1}{2}$ inch | | | | clear and | |
| " M. | 1 inch | medium | medium | northerly | dry | |
| " 7 P. M. | $\frac{1}{2}$ inch | | | | | 11 |

| Date. | Length of electrical spark. | Indication S. side of building. | of Ozone, N. side of building. | Winds. | Weather. | Mortality. |
|---|---|---|---|---|---|---|
| Aug. 5, 7 A. M. | none | | | southerly | rain and | |
| " M. | none | dark | dark | north | thunder | |
| " 7 P. M. | none | | | | | 12 |
| Aug. 6, 7 A. M. | $\frac{1}{2}$ inch | | | | | |
| " M. | 2 inch | medium | light | northerly | clear and | |
| " 7 P. M. | 1 inch | | | | dry | 7 |
| Aug. 7, 7 A. M. | $\frac{1}{2}$ inch | | | | dry | |
| " M. | $\frac{1}{4}$ inch | dark | medium | southerly | cloudy and | |
| " 7 P. M. | $\frac{1}{8}$ inch | | | | damp | 14 |
| Aug. 8, 7 A. M. | none | | | | | |
| " M. | none | dark | dark | | damp,cloudy | |
| " 7 P. M. | $\frac{1}{4}$ inch | | | southerly | clear | 6 |
| Aug. 9, 7 A. M. | $\frac{1}{2}$ inch | | | | damp | |
| " M. | $\frac{1}{2}$ inch | medium | medium | northerly | | |
| " 7 P. M. | 1 inch | | | | dry | 7 |
| Aug. 10, 7 A. M. | 1 inch | | | | | |
| " M. | $1\frac{1}{4}$ inch | medium | medium | still | clear and | |
| " 7 P. M. | $2\frac{1}{2}$ inch | | | | dry | 13 |
| Aug. 11, 7 A. M. | 2 inch | | | | | |
| " M. | $2\frac{1}{2}$ inch | light | light | northerly | clear and | |
| " 7 P. M. | 2 inch | | | | dry | 9 |
| Aug. 12, 7 A. M. | 2 inch | | | | | |
| " M. | 1 inch | dark | dark | | | |
| " 7 P. M. | $\frac{1}{2}$ inch | | | southerly | cloudy | 8 |
| Aug. 13, 7 A. M. | none | | | | | |
| " M. | none | medium | medium | southerly | rainy | |
| " 7 P. M. | none | | | | | 5 |
| Aug. 14, 7 A. M. | none | | | | | |
| " M. | 1 inch | medium | medium | | dry | |
| " 7 P. M. | 2 inch | | | northerly | clear | 5 |
| Aug. 15, 7 A. M. | 1 inch | | | | | |
| " M. | 1 inch | medium | light | southerly | clear | |
| " 7 P. M. | 1 inch | | | | | 11 |
| Aug. 16, 7 A. M. | $\frac{1}{2}$ inch | | | | | |
| " M. | none | light | light | southerly | damp | |
| " 7 P. M. | none | | | | | 4 |
| Aug. 17, 7 A. M. | none | | | | | |
| " M. | none | medium | medium | northerly | damp and | |
| " 7 P. M. | none | | | | rainy | 7 |
| Aug. 18, 7 A. M. | none | | | | | |
| " M. | none | dark | dark | northerly | damp | |
| " 7 P. M. | none | | | | | 2 |
| Aug. 19, 7 P. M. | none | | | | | |
| " M. | none | dark | dark | southerly | | |
| " 7 P. M. | none | | | northerly | rainy | 5 |
| Aug. 20, 7 A. M. | none | | | | damp | |
| " M. | $\frac{1}{2}$ inch | light | light | northerly | dry | |
| " 7 P. M. | $\frac{1}{4}$ inch | | | | | 3 |
| Aug. 21, 7 A. M. | $\frac{1}{8}$ inch | | | | | |
| " M. | $\frac{1}{2}$ inch | medium | medium | | | |
| " 7 P. M. | none | | | southerly | damp | 1 |
| Aug. 22, 7 A. M. | none | | | | rained all n't | |
| " M. | none | light | light | southerly | | |
| " 7 P. M. | none | | | northerly | rainy | 3 |

| *Date.* | Length of electrical spark. | Indication of Ozone, S. side of building. | N. side of building. | *Winds.* | *Weather.* | *Mortality.* |
|---|---|---|---|---|---|---|
| Aug. 23, 7 A. M. | none | | | | | |
| " M. | none | medium | medium | southerly | damp | |
| " 7 P. M. | none | | | | | 5 |
| Aug. 24, 7 A. M. | none | | | | | |
| " M. | none | dark | dark | northerly | clear | |
| " 7 P. M. | none | | | | | 4 |
| Aug. 25, 7 A. M. | none | | | | | |
| " M. | none | dark | medium | southerly | dry | |
| " 7 P. M. | none | | | | | 2 |
| Aug. 26, 7 A. M. | none | | | | | |
| " M. | none | medium | medium | southerly | rainy | |
| " 7 P. M. | none | | | | | 5 |
| Aug. 27, 7 A. M. | none | | | | damp | |
| " M. | ⅛ inch | medium | medium | | | |
| " 7 P. M. | none | | | northerly | dry | 3 |
| Aug. 28, 7 A. M. | ⅛ inch | | | | | |
| " M. | none | medium | medium | northerly | dry | 0 |

In this table it will be oserved that during the greatest mortality the spaak was the longest, and during the decline of the disease, the manifestation of Electricity was very much diminished.

There was considerable variation in the appearace of the ozone tests, but there is no relation between them and the prevalence of cholera as shown by the mortality.

The winds were mostly from the North or South, and the weather was the best while the disease was the worst.

*Ozone.*---As to Ozone, I have not seen the undeniable evidence of its existence. I am by no means certain that the decomposition of Iodide of Potassium, which, when mixed with starch forms the the test for Ozone, is not the result of the action of the atmosphere modified by light, heat and moisture. Be this as it may, it would seem that the observations given in the foregoing table, and those made at Naperville, and at Jackson, Mich., would indicate that it has but little to do with cholera.

*Boundaries.*---It is still gravely asserted that cholera has an affinity for limestone districts, and a tendency to spare the regions of granite; but these, like a host of other conclusions, are drawn from insufficient data. That granite regions will always be partially exempt from the disease is probable, at least until high and craggy mountains shall impose no barrier to human intercourse, and become elligible sites for great commercial cities. But the prevalence of the disease at Staten Island, a granite formation, on the Green Mountains of Vermont, at Boston, Lowell, and various other places on the regions of primative rock would at once destroy the distinction.

Cholera is subject to no boundaries except those that prevent human intercourse. We find it in the densely populated city, on the dreary desert, and wild western prairie. Laying low the inhabitants of the princely mansion, the cottage, and the Indian wigwam. Traversing all climates, from the scorching Indias, to the regions of perpetual snow; and extending from the Celestial Empire of the East, to the golden valleys of California in the West.

Attacking alike the indigent and the affluent; the filthy and the cleanly; the reckless debauchee and the prudent valetudinarian. The young and the old, the black and the white, the yellow and the red man. In the dreary home of the latter on the extensive plains that stretch out from the Missouri river to the Rocky Mountains, it is now committing its fearful ravages. And as if to put the blush upon our boasted knowledge and philosophy—our enlightened mode of investigation—our learned and logical reasoning—from his own observation he announces to his comrades what its history indicates as the true mode of its propagation, and commences to take vengeance upon the white man for bringing it among them.

